# Biomimetic Approach to CO_2_ Reduction

**DOI:** 10.1155/2018/2379141

**Published:** 2018-08-01

**Authors:** Ilaria Gamba

**Affiliations:** Universidad de La Laguna, San Cristóbal de La Laguna, Santa Cruz de Tenerife, Spain

## Abstract

The development of artificial photosynthetic technologies able to produce solar-fuels from CO_2_ reduction is a fundamental task that requires the employment of specific catalysts being accomplished. Besides, effective catalysts are also demanded to capture atmospheric CO_2_, mitigating the effects of its constantly increasing emission. Biomimetic transition metal complexes are considered ideal platforms to develop efficient and selective catalysts to be implemented in electrocatalytic and photocatalytic devices. These catalysts, designed according to the inspiration provided by nature, are simple synthetic molecular systems capable of mimic features of the enzymatic activity. The present review aims to focus the attention on the mechanistic and structural aspects highlighted to be necessary to promote a proper catalytic activity. The determination of these characteristics is of interest both for clarifying aspects of the catalytic cycle of natural enzymes that are still unknown and for developing synthetic molecular catalysts that can readily be applied to artificial photosynthetic devices.

## 1. Introduction

The employment of devices based on artificial photosynthetic technologies may be the most convincing strategy for generating enough clean energy to satisfy the needs of the expanding world [1, 2]. However, the development of such devices is not an easy task. Different fundamental components, each one committed to a specific function, must be matched together to mimic the ultimate functionality of the natural photosynthetic apparatus [3]. Thus, inspired by natural photosynthesis, the crucial components of an artificial photosynthetic device should be a light harvester for the absorption of sunlight and the generation of charge-separation (e.g., semiconductor or molecular dye) and a reduction active reaction site and an oxidation active reaction site, where the redox processes occur [4]. Such a design is represented by the so called “hybrid photocatalytic” devices, in which semiconductors are employed as light harvesters and transition metal complexes as molecular catalysts to assist specific redox reactions: the carbon dioxide reduction and the water oxidation [5–7]. Indeed, the setting exploited for hybrid photocatalytic devices presents significant advantages. The use of semiconductors as light harvesters, in fact, ensures a broad spectral absorption associated with a good robustness of the materials. Also, the involvement of specific transition metal complexes as catalysts assists and facilitates both the occurrence of redox reactions and the generation of charge separation through fast charge transfers between the semiconductor and the molecular complexes. In particular, the coupling between semiconductors and biomimetic complexes (i.e., hydrogenase, formate dehydrogenases, and carbon monoxide dehydrogenases mimics) may reveal to be an efficient strategy for aiding the CO_2_ reduction, improving the current solar fuel technologies and stimulating the applications of these devices.

Besides the development of artificial photosynthetic devices, aimed to stimulate “solar fuels” production, the investigation focused on the activation and conversion of CO_2_ is also of main interest to improve the strategies nowadays used for the treatment of its constantly increasing emissions [8]. Indeed, the rising of CO_2_ emissions has led to serious greenhouse effects and global warming, facing the necessity to adopt CO_2_ conversion technologies, able to transform this greenhouse gas into value-added products, to ensure an equilibrate global carbon cycle, and to mitigate the global warming through the reduction of atmospheric CO_2_ [9].

However, the CO_2_ activation, reduction, or conversion is not easy to accomplish. CO_2_ is a thermodynamically stable molecule and kinetically inert. Indeed, the electrocatalytic CO_2_ reduction demands high over-potentials to occur and results in poor selectivity of product formation (mixtures of CO and formate are generally obtained). Furthermore, CO_2_ reduction is typically characterized by low faradaic efficiency due to the competition with the hydrogen evolution reaction (HER), which takes place at the same range of potentials [10]. In this context, the development of novel, specific, and efficient catalysts to perform CO_2_ activation, reduction, or conversion is nowadays a major challenge to be faced. Transition metal compounds can be used as ideal platform to realize these tasks, both considering homogeneous and heterogeneous (thus, associated to an electrode) applications [11–14].

The present review aims to focus the attention on the progresses achieved employing transition metal complexes, designed according to the inspiration devoted to the bioinorganic approach, as catalysts to promote carbon dioxide (CO_2_) two-electron reduction. Furthermore, the active site structures and the mechanistic features associated with the CO_2_ reduction activities of natural enzymatic systems (in particular carbon monoxide dehydrogenases, CODH, and formate dehydrogenases, FDH, and enzymes) are herein described, with the aim of highlighting fundamental principles of their efficient catalytic activity. These aspects are of main importance to inspire the design of simple synthetic molecular systems able to reproduce the enzymes functionality.

Moreover, the development of electrocatalysts, able to perform the two-electron reduction of CO_2_ to CO and/or formate, closely related to the enzymatic activity, are here listed. Particular attention is paid to the description of proposed reaction mechanisms and the general structural features that can dictate the selectivity of the products generated from CO_2_ reduction. On the other hand, photoelectrocatalytic systems, which represent attractive alternatives to develop efficient catalysts, [12, 15], were not included in the present discussion.

## 2. CO_2_ Activation and Reduction by Enzymes and Transition Metal Complexes

Nature is widely known to be a source of efficient catalysts: the enzymes and metalloenzymes, which are natural machines that can be esteemed for performing extremely difficult chemical processes with elegant selectivity, fast rates, and low activation energies. Furthermore, metalloenzymes present the advantage of being constructed exclusively from copious bioavailable materials and typically employ only metals abundant in the Earth's crust, such as first row transition metals [16, 17].

However, when considering applications that require sustainability and scalability, enzymes have the major drawback of being quite unstable and work normally only over the limited range of conditions in which the proteins remain correctly folded. Thus, they are not suitable for use directly in all applications, especially when compared to synthetic compounds that can mimic enzymes structure and functionality. All in all, if not directly considered for applications, enzymes serve as inspiration to discover fundamental principles of fast catalysis that can be executed by simple synthetic molecules. In particular, the enzymes that have inspired the development of biomimetic synthetic catalysts for CO_2_ reduction are the carbon monoxide dehydrogenases and the formate dehydrogenases [18, 19].

Key structural and mechanistic features related to these enzymes are herein summarized in the following paragraphs, included in the section dedicated to “carbon dioxide reduction enzymes.”

## 3. Carbon Dioxide Reduction Enzymes

### 3.1. Carbon Monoxide Dehydrogenases (CODHs)

There are two types of known carbon monoxide dehydrogenase (CODH) enzymes, which are the MoCu-CODHs and the Ni-CODHs, respectively, characterized by different metals (MoCu or Ni) contained in their active sites [18]. Both enzymes are able to catalyze the oxidation of CO to CO_2_, albeit Ni-CODHs upkeep the process at much higher turnover frequencies (*k*_cat_ values: 4 × 10^4^ s^−1^) than MoCu-CODHs (100 s^−1^). Under certain circumstances, CO_2_ reduction activity has also been observed in case of Ni-CODHs (although with lower turnover frequencies compared to CO oxidation, approximately 45 s^−1^) but never detected to date for MoCu-CODHs [20, 21].

**Figure 1:**
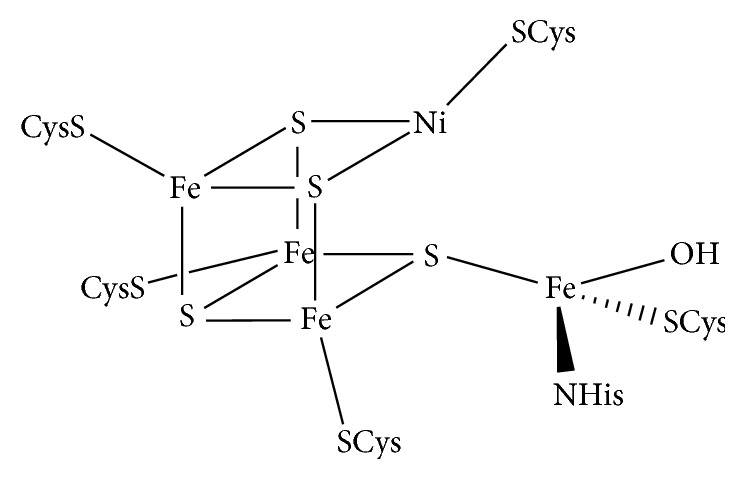
Molecular structure of the C-cluster contained in the Ni-CODH enzymes-active site.

Structural and mechanistic aspects, related in particular to Ni-CODHs, are of main interests also for the development of synthetic catalysts. However, the extreme sensitivity of Ni-CODHs to the presence of dioxygen (indeed, they are found only in anaerobic bacteria) made difficult mechanistic investigation and structural study. In fact, organisms growth, purification, and all manipulation related to these enzymes require definitely strictly anaerobic and often low temperature conditions [22]. Despite these difficulties, the X-ray structures of five Ni−CODHs have been solved to date [23–33], clearly showing the presence of different types of metal clusters: [NiFe_3_S_4_], called the C-clusters, and [Fe_4_S_4_], called B- and D-clusters [19]. The reversible CO/CO_2_ conversion is believed to occur at the C-clusters [22, 34, 35], whereas the B- and D-cluster would be part of the electrons-transporting chain of the protein system. The structure of these C-clusters can be described as a distorted [Fe_4_S_4_] cluster with a nickel atom replacing one of the iron atoms and an iron atom pendent to the cluster, called the “dangling iron” (Figure 1). The metal atoms, which coordinate to cysteine residues and inorganic sulfide ligands are ordinated in a cubane structure, like the one described for [Fe_4_S_4_] clusters. The dangling iron of the [NiFe_3_S_4_] clusters is coordinated by a cysteine and a sulfide ligand of the cubane structure and by a histidine and a hydroxyl ligands (Figure 1) [28].

Four distinct oxidation states of the C-cluster were identified on the basis of spectroscopic studies: C_ox_, C_red1_, C_int_, and C_red2_. C_ox_ (Ni^2+^, Fe^3+^) is an oxidized state of the cluster considered inactive. C_red1_ (Ni^2+^, Fe^2+^) and C_red2_ (Ni^0^, Fe^2+^), which have one and three electrons more reduced than C_ox_, respectively, are proposed to be the active states of the enzyme, directly involved in the catalytic cycle of reversible CO/CO_2_ conversion. Finally, C_int_ (Ni^+^, Fe^2+^) is described as the intermediate state between C_red1_ and C_red2_, thus, two electrons less than C_ox_, and it seems to be an inactive state as well as C_ox_ [28].

**Figure 2:**
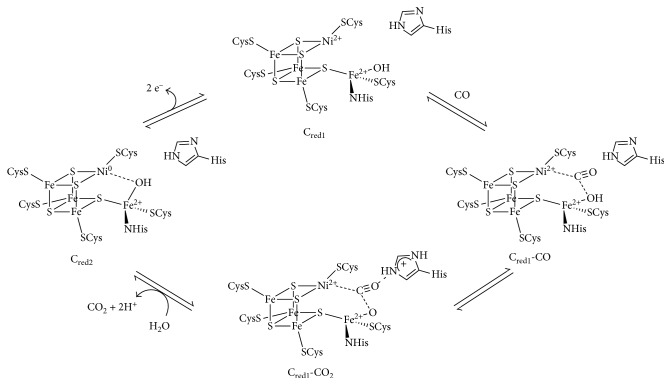
Proposed mechanism for the reversible CO oxidation mediated by Ni-CODH enzymes [18, 19, 28]. Reproduced with permission from [18]. Copyright 2015 Springer Nature.

The catalytic mechanism proposed, for the reversible CO/CO_2_ conversion assisted by Ni−CODHs, suggests that the reaction site should be located between the Ni atom and the dangling Fe atom. Thus, during the first step of CO oxidation (as depicted in Figure 2), the Ni atom is the center responsible for the initial CO binding, when the cluster is in the C_red1_ state. Upon CO binding, the C-cluster undergoes reduction to the C_red2_ state and then transfers the electrons through the B- and D-clusters in the enzyme, generating the intermediate C_red1_-CO state. Afterwards, the hydroxyl group, which is coordinated to the dangling Fe atom, attacks the bounded CO molecule at the carbon atom, leading to the C_red1_-CO_2_ form. At this stage, upon binding of water, a CO_2_ molecule and a proton are released, reducing the C-cluster at the C_red2_. The C-cluster starting state, C_red1_, is recovered upon oxidation of C_red2_ mediated by the B- and D-clusters. Finally, the presence of a histidine residue located in the protein near to the C-cluster is proposed to facilitate the transfer of protons during the reaction and stabilize the intermediate steps providing hydrogen bonds to the coordinated CO and CO_2._

### 3.2. Formate Dehydrogenase (FDH)

**Figure 3:**
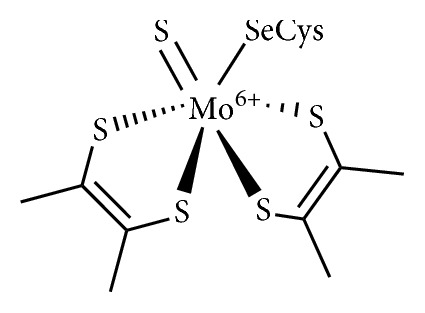
Molecular structure of Mo-dependent FDH enzymes-active site.

The metal-dependent formate dehydrogenase (FDHs) enzymes are able to reversibly catalyze the two-electron reduction of CO_2_ to formate. Between different classes of known FDH enzymes, the NAD^+^-independent FDHs are the ones recognized as responsible for CO_2_ reduction reaction. Like the Ni-CODHs, the NAD^+^-independent FDHs are also extremely sensitive to atmospheric dioxygen, and their manipulation also requires strictly anaerobic conditions. The structures of several of these enzymes were resolved (by a combination of both X-ray crystallographic and EXAFS measurements) and show the presence in their active sites of molybdenum or tungsten atoms, displaying common active sites architecture [36]. According to these data, the reaction site can be described by Mo (or W) center in a trigonal-bipyramidal geometry coordinated by two pyranopterins, one Se-cysteine residue, and one sulfido ligand (Figure 3).

**Figure 4:**
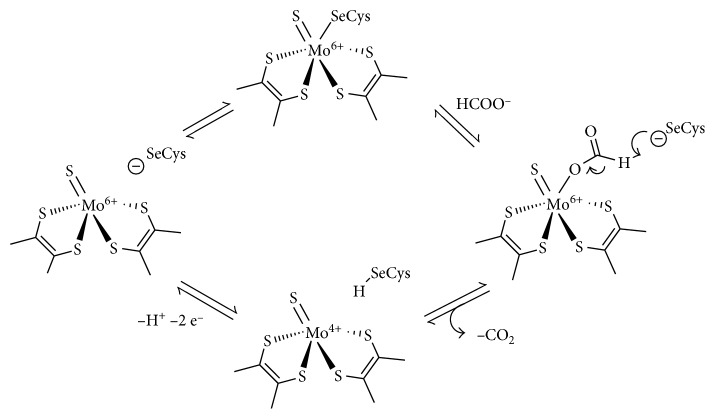
Early mechanistic proposal for the FDH-catalyzed reversible formate oxidation [36, 37]. Reproduced with permission from [37]. Copyright 2006 Springer Nature.

The mechanism proposed for the FDH-catalyzed formate reversible oxidation is currently still under discussion. In case of Mo-FDHs, the Mo atom contained in the active site would assume during the catalytic cycle the Mo(VI), Mo(V), and Mo(IV) formal oxidation states, also supported by the contribution of the redox noninnocent ligand pyranopterin. In this scenario, the Mo(VI) state is responsible for formate oxidation and the Mo(IV) state is used for CO_2_ reduction [36]. Early attempts to propose a reaction mechanism envisioned the possibility of generating of a vacant coordination site, meant for formate binding, upon Se-cysteine dissociation (Figure 4). Thus, initially formate binds at the Mo(VI) center (oxidized form) in the vacant site generated by the released of the Se-cysteine. Subsequently, the *α* proton of the formate is removed, probably by the nearby selenide anion. Reduction of the Mo(VI) to the Mo(IV) state is finally associated with CO_2_ release. The catalytic cycle closes with the regeneration of the initial Mo(VI) state, upon two-electron oxidation of the reduced Mo(IV) form (electrons are transferred from Mo(IV) via a [4Fe–4S] cluster center to an external electron acceptor) and deprotonation of the Se atom that recover its coordination at the metal center [37].

**Figure 5:**
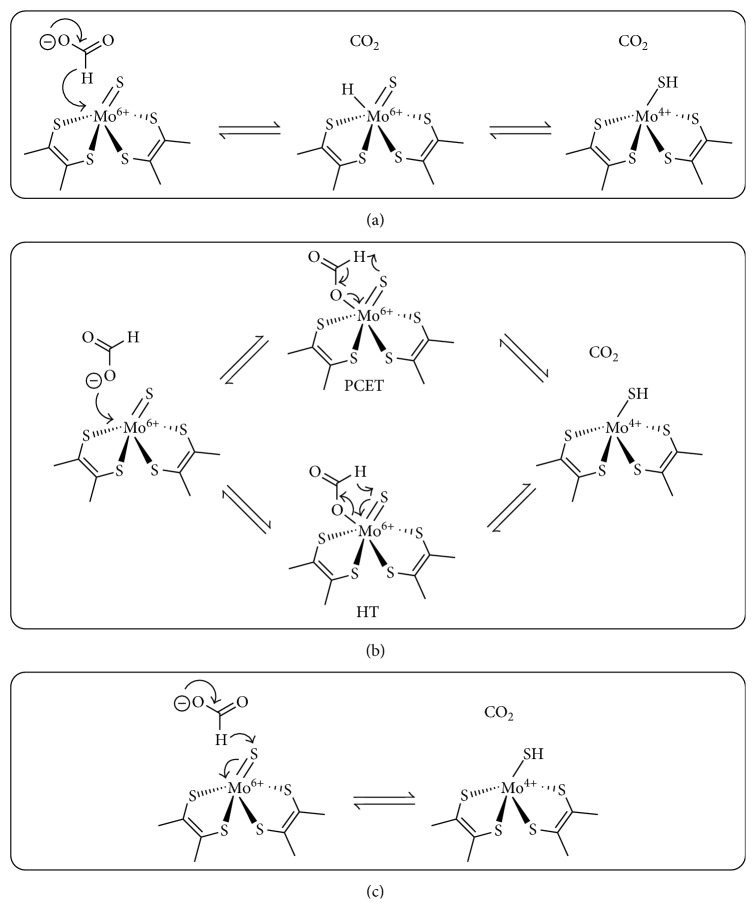
Proposed reaction mechanisms by (a) Tiberti et al. [38], (b) Robinson et al. [36], and (c) Niks et al. [40]. Reproduced with permission from [36]. Copyright (2017) American Chemical Society.

Another suggested panorama for the formate oxidation by metal-dependent FDHs pictures the direct hydride transfer to Mo(VI), followed by hydride migration to the sulfur [38] (Figure 5, mechanism A).

More recently, Reisner and Hirst have suggested a mechanistic proposal supporting that the formate oxidation occurs through a five-membered transition state. In this scenario, formate coordinate to the Mo(VI) by its oxygen lone pairs and then, upon formate oxidation, CO_2_ and Mo(IV)-SH products are generated. The five-membered transition intermediate can either undergo proton-coupled electron-transfer (PCET) reaction or as a hydride-transfer (HT) reaction (Figure 5, mechanism B) [36].

Besides these formulations that picture the dissociation of the Se-cysteine ligand, a scenario, in which the saturated coordination environment at the Mo atom is preserved, has been also considered [39, 40]. In this latter case, the direct hydride transfer of the formate *α*-hydrogen to the terminal sulfido group of the Mo(VI)=S moiety, without substrate binding at the metal, is proposed (Figure 5, mechanism C) [40].

## 4. Carbon Dioxide Reduction Mediated by Synthetic Catalysts

### 4.1. CO_2_ Coordination Modes at Metal Centers

The transformation of carbon dioxide mediated by transition metal catalysts is a research area that is captivating the attention of the scientific community [11, 41, 42]. Diverse products, including CO, formate, methanol, and methane, can be derived from CO_2_ reduction catalyzed by transition metal complexes. In this context, a recent review published by Grice details and collects the more recent achievements in the field of CO_2_ reduction with homogeneous early transition metal complexes (group 3–7). The review focuses the attention on the stoichiometric and catalytic reductions to formate, methanol, and similar products, which have been more frequently observed when early transition metal complexes are employed as catalysts [42]. Besides this report, several publications recently appear highlighting the opportunities to take advantage of transition metal complexes to reduce CO_2_ and to convert it in value-added products [43–47]. For most of these transformations, the CO_2_ activation and reduction is initiated by its coordination at a metal center.

The regulation of the CO_2_ binding mode at metal complexes is of major importance for the development of catalysts that can selectively reduce CO_2_, because it can dictate the distribution of the reaction products [45, 48, 49]. In this regard, a recent reevaluation of known CO_2_-metal complex adducts displays the presence of several binding modes, allowing their complete and accurate classification, as well as the rationalization of the oxidation states assumed by carbon and metal atoms involved in the CO_2_ binding [50].

**Figure 6:**
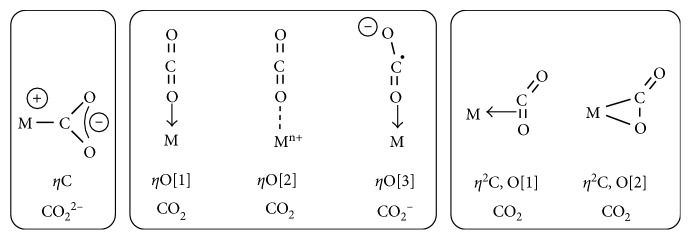
Possible binding modes of CO_2_-monometallic complexes. Reproduced with permission from [50]. Copyright (2017) Elsevier.

The possible CO_2_ metal binding modes are categorized according to the number of directly bonded metal centers and depending on the connectivity (how the atoms are attached to one another). The principal adducts derived from the simplest CO_2_ monometallic complexes are summarized in Figure 6.

At a mononuclear transition metal center, CO_2_ can either coordinate through the C or the O atom, where *η*^O^ exhibits a richer variety of coordination possibilities than the *η*^C^, including *η*^1^ and *η*^2^-(C,O) side-on binding fashions.

The *η*^1^-binding mode is represented by the coordination of one of the CO_2_ oxygen atom at the metal center. Different types of *η*^1^-binding modes can be distinguished depending on the kind of interaction that determines the nature of the binding. On the basis of these data, it is possible to define the existence of *η*^1^- [1], *η*^1^- [2], and *η*^1^- [3] adducts. In case of *η*^1^- [1], CO_2_ datively bound through one of its oxygen atoms to the metal centers. *η*^1^- [2] adducts are characterized by metal-O interaction of electrostatic nature, involving the positively charged metal and the induced negative quadrupole moments at the CO_2_ oxygen atom. Finally, *η*^1^- [3] adducts represent the patterns in which the binding situation is better described by the interaction developed between the one-electron oxidize metal center and the reduced CO_2_^−^ ligand, where the metal would have a positive charge and a negative charge would be located at the non-coordinated oxygen atom. The radical carbon would form a double bond to the datively coordinated oxygen atom (Figure 6).

Besides, the CO_2_ binding mode of *η*^2^-(C,O) side-on adducts can be described by two limiting situations: one in which CO_2_ binds by *π*-donation to the metal, identified as *η*^2^-(C,O) [1], and another in which the bound order of the double C=O bond is reduced as results of the *π*-back donation of the metal center, with concomitant formation of metal-C and metal-O bonds, recognized as *η*^2^-(C,O) [2] (Figure 6).

Cases in which the CO_2_ binding is shared between two, three, or four metal atoms result in an even wider variety of interaction types. A detailed description of these situations can be found in the recent review published by Paparo and Okuda [50].

### 4.2. Structural Models of CODHs

Attempts to mimic the Ni-CODH active sites have been focused either on the preparation of monometallic Ni and heterobimetallic Ni-Fe complexes or on the construction of [NiFe3S4] clusters.

**Figure 7:**
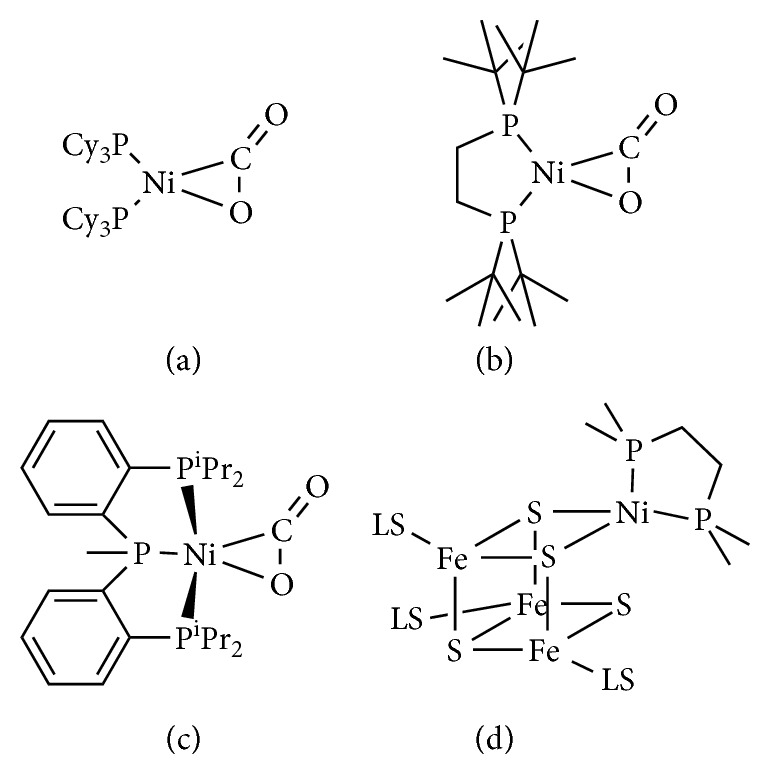
Molecular structure of the Ni-based catalysts reported by (a) Aresta et al. [51], (b) Hillhouse et al. [53], (c) Lee et al. [54], and (d) Holm et al. [55].

Few examples appear in the literature describing mononuclear Ni-CO_2_*η*^2^-adducts, which present spectroscopic characteristics in close relation to the Ni center of the C-cluster found in Ni-CODHs enzymes. The first structurally characterized Ni-CO_2_*η*^2^-adduct, ((PCy_3_)_2_Ni(*η*^2^-CO_2_)), was reported in 1975 by Aresta and co-workers, [51, 52], followed by the more recent example presented by Hillhouse, the (dtbpe)Ni(*η*^2^-CO_2_) (dtbpe = 1,2-bis(di-tert-butylphosphino)ethane) adduct [53] (Figures 7 and 7). Both the compounds are based on tetracoordinated nickel centers, with two phosphorus donor atoms and one *η*^2^-CO_2_ ligand.

More recently, another interesting Ni-CO_2_*η*^2^-adduct was structurally characterized by Lee et al., the (PP^Me^P)Ni(*η*^2^-CO_2_) adduct (where PP^Me^P is the PMe[2-P^i^Pr_2_-C_6_H_4_]_2_ ligand, Figure 7) [54]. In this latter case, the Ni center is 5-coordinated by three phosphorus donor atoms and one CO_2_ ligand in a *η*^2^-binding mode. Besides the same group, taking advantage of the similar but anionic PNP ligand (PNP^−^ = -N[2-P^i^Pr_2_-4-Me-C_6_H_3_]_2_) reported the synthesis and characterization of novel nickel-carboxylate adducts. The carboxylate ligand, at the Ni center of these compounds, is stabilized by Lewis acids, such as a proton (nickel hydroxycarbonyl, (PNP)NiCOOH), sodium (nickel sodium carboxylate, (PNP)NiCOONa), or another metal ion.

Using this strategy, also a dinuclear nickel-iron carboxylate species, (PNP)Ni-*μ*-CO_2_-*κC*:*κ*^2^*O*,*O′*-Fe(PNP), was prepared and characterized [56]. This latter complex represents the first example of nickel-iron hetero-bimetallic complex equipped with a bridging CO_2_ ligand, structurally reminiscent of the NiFe-binuclear active site of CODH enzymes.

In parallel to these studies, the synthetic effort spent for mimicking the unique [NiFe_3_S_4_] core structure resulted in the biomimetic compounds reported by Holm and coworkers, which represent the closest synthetic analogues of the C-cluster found in anaerobic CODHs [55, 57–59]. The approach used satisfies fundamental structural requirements of the CODHs-active site modeling, as the incorporation of a Ni atom at the apex of an iron-sulfur cubane [57]. Also, rational synthetic modifications forced the planarization of the tetrahedral nickel center, upon breaking of one Ni-S bond, attaining square planar Ni(II) in a Ni-Fe-S cubanoid cluster, as owned by the enzyme (Figure 7) [55, 58]. Unfortunately, the presence of a pending iron atom in a synthetic complex was not ever realized. Neither of these compounds, however, was found able of promoting CO_2_ reduction or CO oxidation activities.

### 4.3. Functional Models for CODHs: Electrocatalytic CO_2_ Reduction to CO Mediated by Molecular Complexes

A great research effort was dedicated to the development of molecular catalysts able to act as CODHs functional models. The transformation of CO_2_ in solution and in particular its reduction to CO through the assistance the of transition metal complexes as electrocatalysts have been comprehensively studied and reviewed [42, 45, 60–65]. These compounds have no structural relation with the active site of the CODHs and cannot be considered structural mimics of these enzymes. However, metal electrocatalysts can reproduce the CODHs activity of CO_2_ reduction to CO, thus resulting in fundamental interests to elucidate aspects of the catalytic mechanism of these enzymes that are still unknown.

In this context, the catalytic model systems more closely related to the CODHs activity are herein described by detailing in the following paragraphs the most representative examples of transition metal compounds based on phosphine or macrocyclic ligands.

#### 4.3.1. Transition Metal Complexes Bearing Phosphine Ligands

Transition metal phosphine complexes have crucially contributed to the understanding of the possible intermediates involved in the CO_2_ reduction reactions. In this context, palladium complexes bearing polydentate phosphine ligands, of the type [Pd(triphosphine)(solvent)]^2+^, represent the most extensively studied catalysts for the electrocatalytic CO_2_ reduction.

The mechanism of this class of catalysts, firstly reported by DuBois and coworkers [66, 67], has been studied in detail [18, 45, 49, 61, 68, 69].

**Figure 8:**
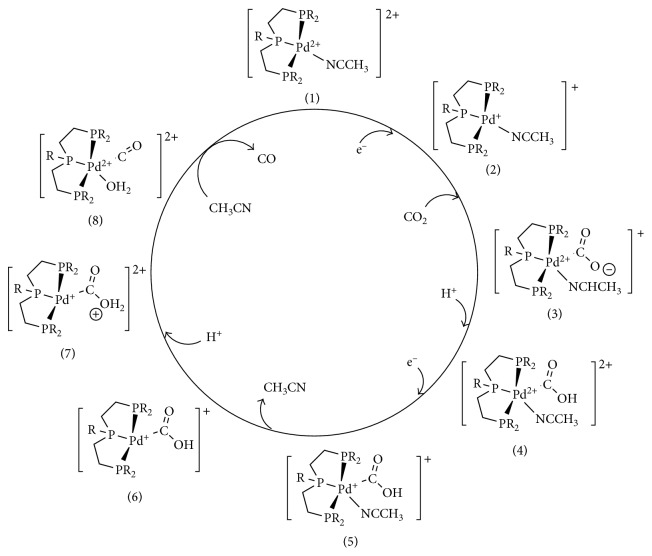
Proposed catalytic cycle for the Dubois Pd catalyst [49, 61]. Reproduced with permission from [49]. Copyright (2009) American Chemical Society.

As represented in Figure 8, the proposed catalytic mechanism began with the Pd(II) **(1)** electrochemical reduction to Pd(I) **(2)**, performing the reaction in DMF and in presence of acid. Following this initial reduction, CO_2_ coordinate at the Pd(I) center and the metal-carboxylate intermediate **(3)** is generated upon oxidation of Pd(I) to Pd(II). Subsequent protonation of the carboxylate ligand affords a metallocarboxylic acid intermediate **(4)**. The second electroreduction of Pd(II) to Pd(I) **(5)** causes the release of the coordinated solvent molecule **(6)**. Then, additional protonation of the carboxylic acid ligand allows the formation of ‘‘dihydroxy carbene” intermediate **(7)**. CO would be generated at this intermediate, by dehydration of the dihydroxy carbene species with concomitant oxidation of Pd(I) to Pd(II) **(8)**. The regeneration of the initial catalysts is achieved by solvent coordination and CO release.

The catalytic current observed was found to depend on the acid concentration and the pH dependence observed is consistent with two protonation steps being involved in the transition state [61].

**Figure 9:**
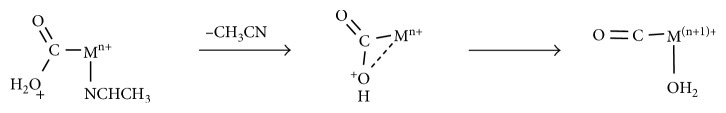
Mechanism proposed for the cleavage of the C-O bond at a metal center. Reproduced with permission from [49]. Copyright (2009) American Chemical Society.

These studies have highlighted the importance of having a weakly bounded molecule coordinated at the metal center in order to develop a proper catalytic activity. Thus, a vacant coordination site would be required for the reaction to occur. In particular, the production of CO from CO_2_, which involves the cleavage of a C-O bond, would be favored by the presence of a vacant coordination site adjacent to the dihydroxy carbene ligand. This specific structural characteristic allows the formation of a metal-O bond upon cleavage of the C-O bond, resulting in the formal migration of a water molecule as depicted in Figures 8 and 9.

The requisite of having a vacant coordination site at a specific position to the CO_2_-bounded ligand, featured in case of these transition metal catalysts, can be found also of biological importance to enlighten aspects of the catalytic mechanism proposed for natural enzymes.

On the other hand, inspired by the bimetallic active site of CODHs, dinuclear analogues of such mononuclear [Pd(triphosphine)(solvent)]^2+^ complexes were designed, with the aim of enhancing the CO_2_-binding properties through the creation of a bifunctional active site [70]. The activity of homobimetallic catalysts was found much improved compared to their monometallic analogues. However, rapid formation of a Pd-Pd bond inactivates the catalyst, resulting in a relatively low TON. Such an inactivation step is prevented in the native Ni-Fe CODH enzymes due to the difference in redox potentials of Ni and Fe centers, which results in a slight tendency to promote metal-metal bound formation.

#### 4.3.2. Transition Metal Complexes Bearing Macrocyclic Ligands

**Figure 10:**
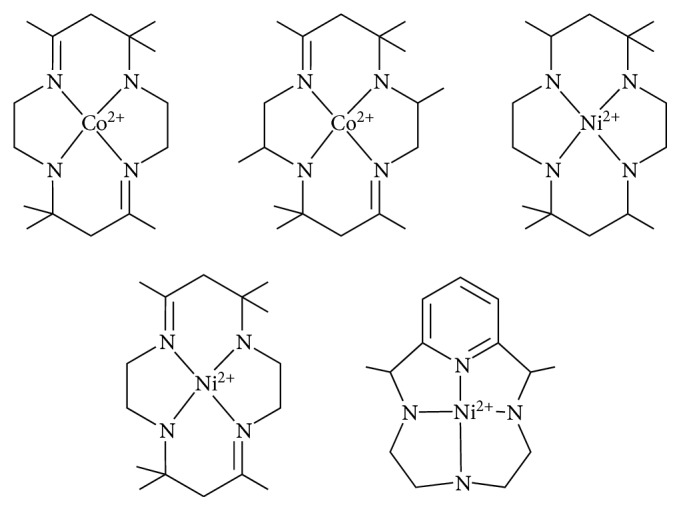
Eisenberg catalysts for the reduction of CO_2_ to CO [71].

*(1) N*_*4*_ *-Macrocyclic Ligands*. The first report about N_4_-macrocyclic transition metal catalysts, which efficiently performed the electrocatalytic reduction of CO_2_ to CO, appeared in 1980 was published by Eisenberg (Figure 10) [71].

In this work, cobalt and nickel N_4_-macrocyclic complexes were demonstrated to be able to reduce CO_2_ to CO, albeit at high overpotentials (ranging from −1.3 to −1.6 V versus SCE). Furthermore, at such potentials, these systems suffer the competing production of molecular hydrogen.

**Figure 11:**
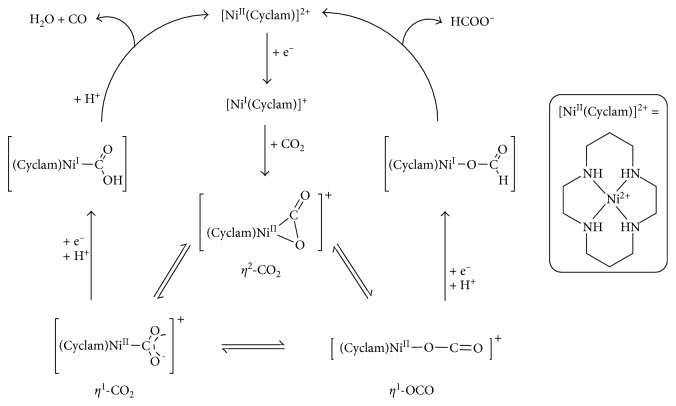
Proposed mechanism for CO_2_ reduction mediated by [Ni(II)cyclam]. Reproduced with permission from [48]. Copyright (2014) American Chemical Society.

More recently, Sauvage and coworkers have extensively studied the reduction of CO_2_ mediated by Ni-cyclam (cyclam = 1,4,8,11-tetraazacyclotetradecane) complexes (Figure 11) [48, 72]. Interestingly, CO_2_ reduction catalyzed by Ni-cyclam complexes, performed in water, leads exclusively to the formation of CO as product. Whereas, using mercury electrodes, CO_2_ conversion mediated by transition metal complexes displays normally the formation of CO together with formate and oxalate as common products [73]. Furthermore, CO_2_ reduction catalyzed by Ni-cyclam complexes, performed in water-DMF (dimethylformamide) mixture, leads to the formation of both CO and formate in different ratio depending on the reduction potential applied.

In the 1980s, Collin and coworkers proposed a reaction mechanism to rationalize how both CO and formate can be generate from CO_2_ reduction mediated by Ni-cyclam complexes (Figure 11) [74–76].

[Ni(cyclam)]^+^ generated by one-electron reduction from [Ni(cyclam)]^2+^ is the species responsible for the initial CO_2_ binding and the active species of the catalytic cycle. The mechanism proposed envisions that the CO_2_ reduction likely proceeds through an inner-sphere electron transfer mechanism. The formation of an adduct, upon CO_2_ coordination to the catalyst, should represent the initial step of the catalytic cycle. However, [Ni(CO_2_)(cyclam)]^+^ has never been trapped at sufficiently high concentration to be fully spectroscopically characterized.

In early study by Collin, it was suggested that CO_2_ can actually bind to [Ni(cyclam)]^+^ through three possible coordination modes, in equilibrium between each others.

The reaction pathway followed after the formation of the initial CO_2_ adduct would be dictated by the adopted binding mode. Thus, upon uptake of an electron and a proton, *η*^1^-CO_2_ species yield to a carboxylate intermediate, [Ni(C(O)OH)(cyclam)]^+^, that subsequently undergoes heterolytic C−O bond cleavage to generate CO in the presence of a proton. Instead, the *η*^1^-OCO complexes evolve into nickel-formate species, [Ni(OCOH)(cyclam)]^+^.

The selectivity for CO generation observed in case of the reaction mediated in water by Ni(cyclam) catalysts was recently elegantly explained by the aid of computational calculations. The formation of *η*^1^-CO_2_ adducts was indeed found energetically favored by 14 kcal/mol compare to *η*^1^-OCO. Thus, under the studied conditions, the reaction pathway proposed to result in formate generation would be blocked [48].

**Figure 12:**
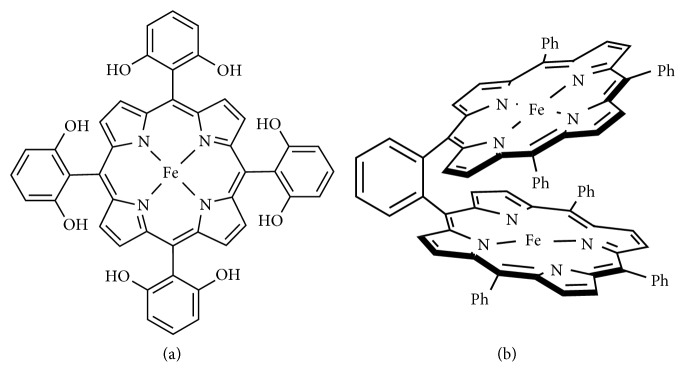
Molecular structure of (a) FeTDHPP and (b) ortho-Fe_2_DTPP complexes.

*(2) Porphyrin Ligands*. A remarkable example of macrocyclic metal complex employed as CO_2_ reduction catalysts is represented by the iron porphyrin FeTDHPP complex, where TDHPP is the 5,10,15,20-tetrakis(2′,6″-dihydroxylphenyl)-porphyrin ligand (Figure 12). FeTDHPP was reported to catalyze the CO_2_ reduction to CO, in acidic dimethylformamide (DMF) solutions, with modest overpotential of less than 0.5 V and 90% faradaic yield for CO production through 50 million turnovers over 4 hours (3500 s^−1^) [77]. The authors suggested that this notable activity could be due to the presence of pendant hydroxyl group that assists the cleavage of the C-O bond by either favoring the proton transfer to the CO_2_ bounded at the metal center or stabilizing the Fe-CO_2_ adduct by hydrogen bounding.

Inspired by the Ni-Fe containing metalloenzymes, cofacial Fe(0) tetraphenyl porphyrin dimers and ortho- and meta-Fe_2_DTPP (Figure 12) were also reported [13, 78]. These compounds were demonstrated to be effective catalysts for CO_2_ electroreduction. The catalysts exhibited excellent selectivity for CO generation and improved faradaic yield (95%) and turnover frequencies (4300 s^−1^), at a moderate overpotential (0.66 V).

**Figure 13:**
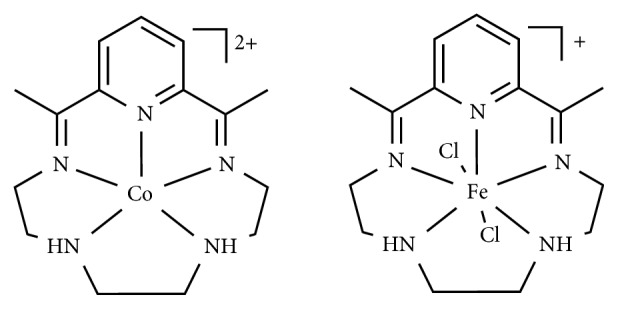
Molecular structure of the cobalt and iron complexes [79].

*(3) N*_*5*_ *-Macrocyclic Ligands*. Robert and coworkers recently reported two novel efficient electrocatalysts, based on cobalt and iron complexes bearing N_5_-macrocyclic ligands (Figure 13) [79].

The electrocatalytic reduction of CO_2_ mediated by the cobalt complex produces CO with high efficiency and selectivity. On the other hand, the process mediated by the Fe complex generates mainly formic acid.

**Figure 14:**
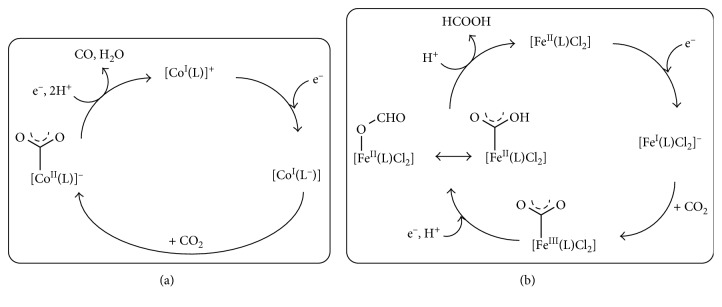
Reaction mechanism proposed for the CO_2_ reduction mediated by cobalt (a) and iron (b) catalysts. Reproduced with permission from [79]. Copyright (2015) American Chemical Society.

The mechanisms proposed for the two different reaction pathways are depicted in Figure 14.

The authors suggested that the selectivity of products formation through the two-electron reduction of CO_2_, CO, or HCOOH could be attributed to distinct protonation and reduction steps for the reactions mediated by cobalt or iron, respectively. The breaking of the C-O bond would be favored at the cobalt-based catalysts (Figure 14(a)), whereas the iron-based catalysts support the formation of a *η*^1^-OCOH intermediate, which would evolve into formic acid release upon protonation (Figure 14(b)).

### 4.4. Functional Models for FDHs: Electrocatalytic CO_2_ Reduction to Formate Mediated by Molecular Complexes

Homogeneous transition metal catalysts were certainly found able to reduce CO_2_ to formate through diverse reaction pathways; for example, as previously mentioned, *η*^1^-OCOH or *η*^1^-OCO intermediates upon protonation would evolve into formic acid or formate release, respectively. In particular, the electrocatalytic systems more closely related to the activity of FDH enzymes actuate the CO_2_ reduction to formate following mechanistic paths that involve the CO_2_ insertion into a metal-H bond.

An important breakthrough in the field of functional FDH enzymes modeling came in 1981, when Darensbourg et al. reported that anionic metal hydrides react readily with CO_2_ to generate metal-formate species [80]. The research of Darensbourg and coworkers showed for the first time that CO_2_ can be inserted into a Ni-H bond of a nickel-triphosphine complex, the trans-[(H)_2_Ni(PCy_3_)_2_], to generate trans-[(H)(HCO_2_)Ni(PCy_3_)_2_] species [81].

**Figure 15:**
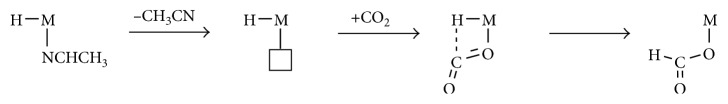
Mechanism proposed for the CO_2_ insertion into a metal-H bond. Reproduced with permission from [49]. Copyright (2009) American Chemical Society.

The presence of two adjacent sites, one destined to coordinate a hydride ligand and the second for CO_2_ binding, likely facilitate C-H bond formation and subsequent formate production, as depicted in Figure 15 [49].

Since the finding by Darensbourg and coworkers, numerous research groups have studied this reaction mediated by transition metal catalysts (especially employing late transition metal as Ru, Rh, Ir, Ni, and Pd).

On the basis of these studies, it was highlighted that the insertion of CO_2_ into the M-H bond could actually occur following two possible pathways.

**Figure 16:**
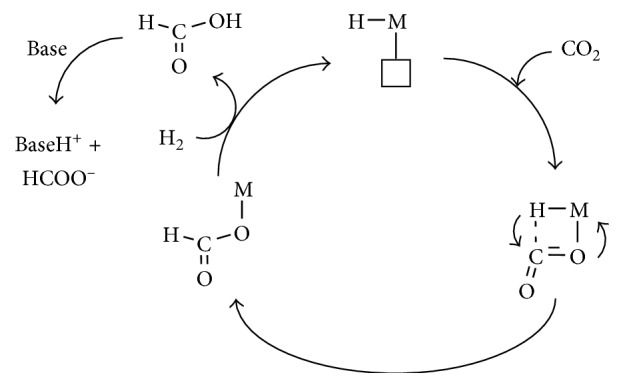
General reaction mechanism describing the catalytic CO_2_ reduction to formate [41]. Reproduced with permission from [41]. Copyright (2011) John Wiley and Sons.

**Figure 17:**
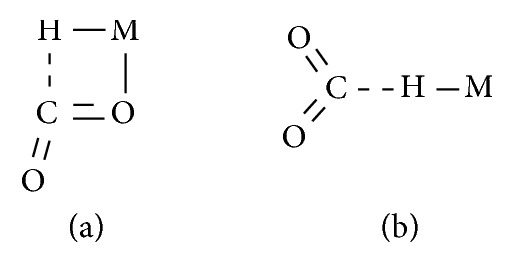
Intermediates proposed for CO_2_ reduction to formate.

Thus, CO_2_ can either coordinate in a *η*^2^-fashion to the metal center and receive the migration of the hydride ligand at the carbon atom or develop a weak H···CO_2_ interaction, which leads to M-H bond cleavage and formate generation (Figures 16 and 17) [41].

The former mechanistic path is the generally accepted (Figure 16 and Intermediate A in Figure 17) [82], while the latter alternative (Intermediate B, Figure 17) has been supported essentially by theoretical studies [83–85].

The addition of a base to generate formate salt (as highlighted in Figure 16) is aimed at facilitating the catalysts regeneration. In fact, precipitation of formate salts allowed the easy separation of products and catalysts.

## 5. Final Remarks

The design of biomimetic transition metal complexes can decisively contributed to elucidate aspects that concern enzymes structure and reactivity. The statement of both the structural and functional characteristics, mandatory to develop a proper enzymatic activity, is critical from a fundamental point of view and may result in key-importance for the construction of synthetic catalysts employable for industrial applications.

As product of this mutual convenience, efficient transition metal catalysts and electrocatalysts, mimicking the enzymatic CO_2_ two-electrons reduction to CO and/or formate, have been developed, and the more significant related examples are reviewed herein. Special emphasis is given to define the structural characteristics that have been highlighted to be compulsory for achieving a fruitful catalytic activity. The development of novel compounds that satisfy these features can lead to their readily application for electrocatalytic devices and to a better understanding of the mechanism of action of both natural and synthetic systems.
